# Impact of maternal death reviews at a rural hospital in Zambia: a mixed methods study

**DOI:** 10.1186/s12939-020-01185-5

**Published:** 2020-07-09

**Authors:** Jean-Bertin Bukasa Kabuya, Arthur Mataka, Gerald Chongo, Luc Kambale Kamavu, Priscilla N’gandu Chola, Christine Manyando, Vincent De Brouwere, Matthew M. Ippolito

**Affiliations:** 1 Department of Clinical Sciences, Tropical Diseases Research Centre, Ndola, Zambia; 2 Saint Paul’s General Hospital, Nchelenge, Zambia; 3 Nchelenge District Health Office, Nchelenge, Zambia; 4 Department of Public Health, Tropical Diseases Research Centre, Ndola, Zambia; 5 Unit of Health Services Organization, Department of Public Health, Institute of Tropical Medicine, Antwerp, Belgium; 6 Johns Hopkins University School of Medicine, Baltimore, MD USA; 7 Johns Hopkins Bloomberg School of Public Health, Baltimore, MD USA

**Keywords:** Maternal health, Hospital epidemiology, Quality improvement, Rural health, Zambia

## Abstract

**Background:**

Maternal mortality in sub-Saharan Africa remains high despite programmatic efforts to improve maternal health. In 2007, the Zambian Ministry of Health mandated facility-based maternal death review (MDR) programs in line with World Health Organization recommendations. We assessed the impact of an [MDR program] at a district-level hospital in rural Zambia.

**Methods:**

We conducted a mixed methods convergent study using hospital data on maternal mortality and audit reports of 106 maternal deaths from 2007 to 2011. To evaluate the overall impact of MDR on maternal mortality, we compared baseline (2007) to late (2010–11) post-intervention inpatient maternal mortality indicators. MDR committee reports were coded and dominant themes were extracted in a qualitative analysis. We assessed potential risk factors for maternal mortality in a before-and-after design comparing the periods 2008–09 and 2010–11.

**Results:**

In-hospital maternal mortality declined from 23 per thousand live births in 2007 to 8 per thousand in 2010–11 (*P* < 0.01). Maternal case fatality for puerperal sepsis and uterine rupture decreased significantly from 63 and 32% in 2007 to 10 and 9% in 2010–11 (P < 0.01). No significant reduction was seen in case fatality due to postpartum hemorrhage. Qualitative analysis of risk factors for maternal mortality revealed four core themes: standards of practice, health systems, accessibility, and patient factors. Specific risk factors included delayed referral, missed diagnoses, intra-hospital delays in care, low medication inventory, and medical error. We found no statistically significant differences in the prevalence of risk factors between the before-and-after periods.

**Conclusions:**

Implementation of MDR was accompanied by a significant decrease in maternal mortality with reductions in maternal death from puerperal sepsis and uterine rupture, but not postpartum hemorrhage. Qualitative analysis of audit reports identified several modifiable risk factors within four core areas. Comparisons of potential explanatory factors did not show any differences over time. These results imply that MDR offers a means for hospitals to curtail maternal deaths, except deaths due to postpartum hemorrhage, suggesting additional interventions are needed. Documentation of MDR meetings provides an instrument to guide further quality improvements.

## Background

Maternal mortality refers to the death of a woman from causes related to or exacerbated by pregnancy or childbirth. Most maternal deaths occur during childbirth or within 48 h postpartum, and is most commonly due to postpartum hemorrhage, puerperal sepsis, hypertensive disorders (e.g. eclampsia) or uterine rupture [1, 2].

Maternal mortality differs vastly between high-income countries and low- and middle-income countries; the latter account for 99% of maternal deaths, and sub-Saharan Africa alone accounts for two-thirds [3]. Decades of concerted global efforts on the part of the United Nations and the multi-institutional Countdown to 2015 partnership to improve maternal health have been met with limited success. Between 1990 and 2015, maternal deaths worldwide decreased by 45%, short of the 75% target set by the Millennium Development Goals [4]. In sub-Saharan Africa, maternal deaths decreased from 990 to 510 per 100,000 live births, remaining over thirty times higher than in developed regions [4]. The partial achievements of the Millennium Development Goals were succeeded by the United Nations Sustainable Development Goals and the Countdown to 2030 partnership, which aims to reduce maternal deaths in every country worldwide to < 140 per 100,000 live births by the end of this decade [5]. To achieve this, guaranteeing access to comprehensive, quality reproductive health services and skilled health professionals at the time of delivery is crucial, as are identifying causal factors surrounding maternal deaths and formulating informed responses [6, 7].

One means by which the latter is accomplished is through facility-based internal reviews of maternal deaths. Maternal death review (MDR) is a decades-old quality improvement approach centered on reviewing and deriving lessons from circumstances surrounding the deaths of individual mothers [8]. In 2004, the World Health Organization (WHO) established technical guidelines for carrying out MDRs to help health officials develop safe motherhood strategies and improve clinical and public health practice, and by 2013 approximately half of the Countdown countries had implemented community- and/or facility-based programs [7, 9]. Yet the evidence for the effectiveness of MDR in resource-limited settings is equivocal [10]; some studies identified MDR-associated improvements in maternal health services or reductions in maternal deaths [11–17], whereas others found no impact [18–20]. Although an old practice, MDR remains an essential component of WHO-recommended maternal death surveillance and response [9], and consolidating evidence of its impact on maternal morbidity and mortality in relevant contexts remains crucial to global safe motherhood strategies.

In 2007, the Ministry of Health of the Government of the Republic of Zambia developed a Reproductive Health Roadmap in effort to reduce maternal mortality [21]. The Zambian experience reflects that of sub-Saharan Africa at large, with maternal mortality of 591 deaths per 100,000 live births in the year the Roadmap was published, and more recent estimates by the WHO of 224 maternal deaths per 100,000 live births in 2015, translating to a lifetime risk of maternal death of 1 in 79 Zambian women [22]. The Reproductive Health Roadmap incorporated WHO recommendations for health facility-based reviews, and Zambia therein joined other Countdown countries in implementing a nationwide MDR program. However, to the best of our knowledge, the impact of MDR on maternal mortality in Zambia and the factors surrounding maternal deaths have not previously been evaluated in rigorous study designs.

We conducted a mixed methods convergent study to assess the impact of facility-based MDR on maternal mortality at a rural, district-level hospital in Zambia with the objective of informing policymakers and stakeholders in maternal health programs. In this setting, women often die of pregnancy-related complications because of delayed care-seeking behavior, or delays in reaching appropriate health facilities [23]. In-hospital maternal mortality is thereby worsened by late presentation to care, in addition to hospital related barriers to adequate obstetric care [23]. We hypothesized that MDR implementation would improve subsequent obstetric care and thereby reduce maternal mortality as problems were identified and MDR committee recommendations were applied to health facility workflow, provider practice, and resource utilization, affecting change across the local health system.

## Methods

### Study design

This was a mixed methods convergent study [24]. Quantitative and qualitative methods were simultaneously applied to assess the impact of a facility-based MDR program on maternal mortality at Saint Paul’s General Hospital, a rural hospital in Luapula Province, Nchelenge District, Zambia [24]. The 175-bed hospital is a referral center for 11 surrounding health clinics, including two island clinics, serving the local district with a population of over 160,000 people and receiving referrals from at least three neighboring districts 90 to 300 km away.

The study subjects included all obstetric patients who died at Saint Paul’s General Hospital due to complications of pregnancy during the study period. Aggregated obstetric outcomes data from hospital registries were collected and analyzed quantitatively to investigate common causes of maternal death and trends in case fatality over time. To identify potential causal factors in the observed patterns of maternal death, and to examine trends over time in these factors, a qualitative analysis of MDR reports of individual patient cases was performed.

### Description of the intervention

In 2007, the hospital developed an MDR program following WHO guidelines. During the first year, a pre-program baseline assessment was performed, and the following year, in 2008, in-depth reviews were initiated. Training was provided by the Provincial Health Office to district and hospital leadership. Meetings were convened typically within 1–2 weeks following a maternal death. They were chaired by a physician (hospital medical officer-in-charge or delegate) and the hospital nursing manager served as the committee secretary. All hospital staff physicians, nurse managers, clinical officers, pharmacy technicians, midwives, and any other staff involved in the care of the deceased were required to attend. When possible, the District Health Office clinical care officer and/or mother and child health coordinator attended the meeting.

Prior to each MDR meeting, a case report was drafted and a maternity death notification form was filed by a midwife or nurse involved in the patient’s care. The patient hospital file, including transfer summary, referral form, and antenatal care card, was attached to the case report. When possible, a family member or other individual accompanying the patient was interviewed according to a semi-structured questionnaire. A typical MDR committee meeting consisted of three elements: case presentation, discussion, and formulation of recommendations. Cases were presented by the midwife and/or nurse who compiled the report. The MDR committee chair directed the discussion, which included an evaluation of community, patient, health system, standard-of-care, and other relevant factors in a root cause analysis. The committee issued recommendations according to its assessment of the case. Meeting minutes were shared with the District Health Office maternal and child health coordinator.

### Data collection and measures

Aggregate data on maternal mortality during the study period 2007–2011 were collected from maternity ward registers and hospital health statistics logbooks maintained by the Health Management Information Systems office. Patient-level data were extracted from MDR meeting minutes and supporting documents for cases that occurred from 2008 to 2011. For cases that occurred in 2007 prior to MDR implementation, only aggregate data were available. All data were extracted and coded by the first author.

Qualitative data collection was through manual review of information contained within MDR reports and extraction of data according to a prespecified rubric containing demographic and clinical variables that was iteratively modified (Appendix 1). Data items were recorded either as present or absent for binary variables (e.g., oxytocin administration, partograph use error) or verbatim from the MDR report for fixed features (e.g. place of delivery, means of transport). Items included demographic information; past obstetric history, including previous access to or not of antenatal care; salient clinical features of the case (e.g. missed diagnosis, partograph use/interpretation, medications administered); and recommendations proposed by the committee. Cases missing data for individual variables were excluded from tabulations, as indicated in Table 1. Missingness was due to the absence of recorded data for that variable; several reports (*n* = 20) did not specify pre-referral care. Validity of qualitative data was established through member-checking among MDR committee members, triangulation of a subset of MDR reports with interviews of patient family members, and peer examination by the first author and co-authors AM, LKK, MMI, and PNC who each have direct patient care experience at the study hospital.

**Table 1:** Characteristics of the study population

Characteristic	No. (%)
Age group	
≤ 19	9 (13)
20–29	22 (33)
30–39	31 (46)
≥ 40	5 (7)
Parity	
0–1	16 (24)
2–4	24 (36)
≥ 5	27 (40)
Length of stay	
≤ 24 h	38 (57)
24–48 h	4 (6)
≥ 48 h	25 (37)
Referred from outside district	26 (39)
Home delivery	9 (13)
Received antenatal care^a^	51 (76)
Used herbal medicines	5 (7)
Delayed care seeking	21 (31)
Delayed ambulance transport^a^	28 (47)
Walked or used public transport	7 (10)
No provision of pre-referral treatment^a^	36 (77)
Cause of death	
Postpartum hemorrhage	14 (21)
Puerperal sepsis	13 (19)
Eclampsia	6 (9)
Uterine rupture	11 (16)
Complication of ectopic pregnancy	2 (3)
Complication of unsafe abortion	1 (1.5)
Malaria-in-pregnancy	5 (7)
HIV/AIDS related complication	10 (15)
Other indirect causes	5 (7)

^a^Data missing for these variables: Received antenatal care, *n* = 1; Delayed ambulance transportation, *n* = 7; No provision of pre-referral treatment, *n* = 20

### Quantitative analysis

Prevalence and standard deviation for maternal deaths were estimated from MDR reports (numerator) and inpatient registers and hospital health statistics logbooks (denominator). We compared overall maternal mortality as a proportion of live births at baseline (2007) to the last two years of the study (late post-intervention period, 2010–11). We also compared case fatality ratios of maternal deaths for the same two periods. We tabulated and examined the proportion of maternal deaths for which one or more of the factors identified in the qualitative analysis contributed to the outcome, comparing the early post-intervention period (2008–09) to the late post-intervention period (2010–11). Differences in proportions were measured using the Pearson’s χ^2^ test for matrices with values ≥5 in all cells or Fisher’s exact test otherwise. All tests were two-tailed and we set a threshold of statistical significance to *P* = 0.05. Data were entered into Epi Info (Version 3.5, Centers for Disease Control and Prevention, USA) and analyzed using Stata (Version 14.0, StataCorps, USA).

### Qualitative analysis

**Fig. 1:**
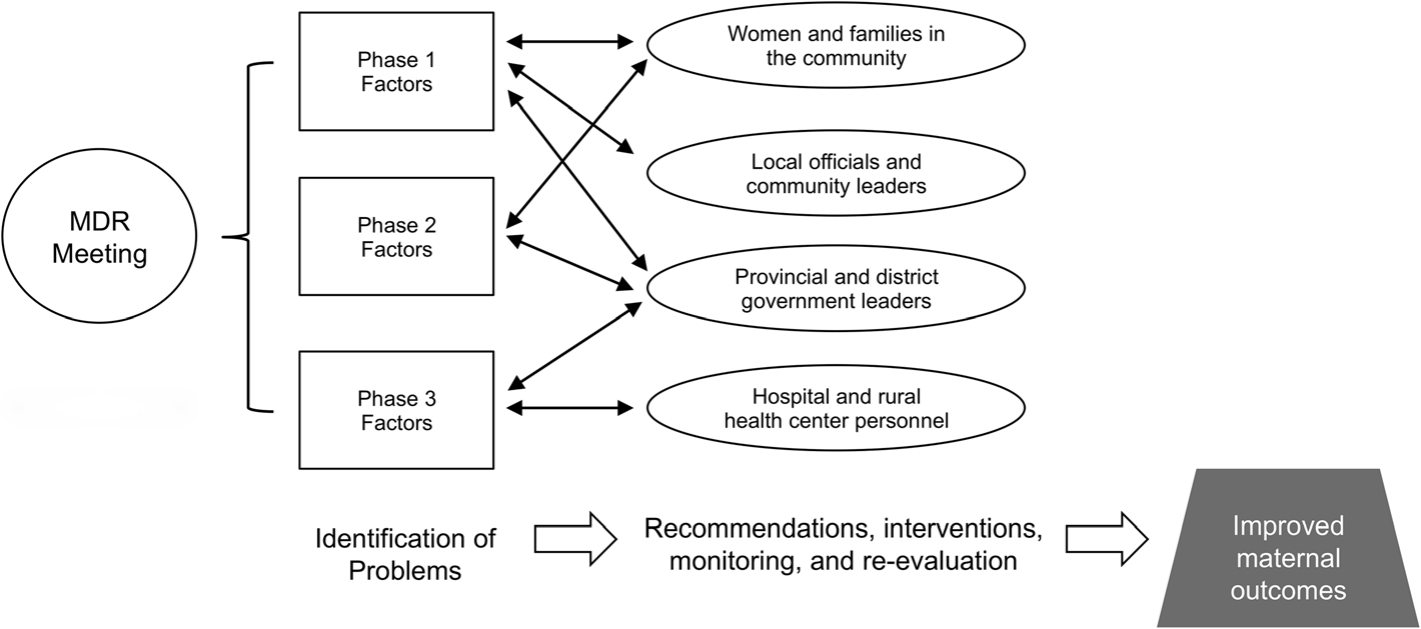
Conceptual framework for the impact of maternal mortality surveillance on quality improvements in obstetric care. Adapted from Thaddeus and WHO maternal mortality or morbidity surveillance models [7, 23]. MDR, maternal death review

We performed directed content analysis and thematic analysis of MDR reports for each identified case [25, 26]. We used thematic analytic procedures to synthesize data and identify opportunities, barriers, and recommendations within a conceptual framework (Fig. 1) based on the Thaddeus Model and WHO Maternal Mortality or Morbidity Surveillance Cycle [7, 23, 24]. The Thaddeus framework categorizes causal factors of maternal death according to the concept of delay [23]. It distinguishes delays in care-seeking behavior on the part of the patient, her family, or both (phase 1); delays in accessing care on account of geographic or other logistical barriers(phase 2); and delays in receiving adequate medical care (phase 3). Phase 3 delays encompass intra- and inter-facility transfer delays, and all aspects of health care provision including clinician skill level, availability of treatments and medical equipment, and other hospital- and provider-level factors. The WHO Maternal Mortality or Morbidity Surveillance Cycle is an iterative process that begins with identification of cases and proceeds through data collection, analysis, recommendations, implementation, evaluation, and refinement [7].

## Results

We identified 106 maternal deaths over the study period. All study subjects had complete MDR reports available. Thirty-nine deaths occurred in 2007, and 67 occurred during 2008–2011 when in-depth reviews were conducted. Characteristics of the latter group are listed in Table 1. The median age was 30 years (interquartile range: 23–44) and most of the women were multiparous (76%). Nearly a third of the women delayed seeking care (31%). A majority of the deaths occurred within 24 h of presentation (57%) and few (13%) had home delivery before reporting to the health facility. The most common cause of death was postpartum hemorrhage (21%) followed by puerperal sepsis (19%), uterine rupture (16%), and eclampsia (9%). Other causes included complications of human immunodeficiency virus (HIV) infection (15%) and malaria (8%). Thematic analysis of MDR committee reports revealed four general categories of opportunities and barriers to care: standards of practice, health systems, accessibility, and patient factors (Table 2).

**Table 2:** Challenges and opportunities identified by case report reviews and family interviews

Core area	Challenges	Opportunities
Standards of practice	*Health Center* • Absent or incomplete screening during antenatal care visits (e.g. hemoglobin, urinalysis, rapid plasma reagin, CD4 testing for mothers with human immunodeficiency virus [HIV] infection) • Late initiation of antiretroviral therapy (ART) among those with HIV infection • Mothers with high risk pregnancies who should have been advised to deliver in a hospital but were not • Measuring and recording of vital signs not done in some cases • Slow to recognize criteria for referral to higher level of care • Preliminary care not provided prior to referral • Incomplete or unclear documentation *Hospital* • Partograph use and/or interpretation errors • Missed or delayed diagnosis • Oxytocin errors • Unnecessary fundal pressure • Antibiotic prophylaxis not given when indicated • Inadequate investigation of postpartum fever • Inadequate wound care • Poor sterilization procedure • Incomplete or unclear documentation	• Routine training by district hospital and Ministry of Health (MOH) of rural health facility providers regarding antenatal screening procedures and approach to high-risk pregnancies, including prevention of mother-to-child HIV transmission • Universal application of partograph monitoring of labor • Ensuring adequate staffing to enable reliable monitoring of contractions when oxytocin is administered • Recruitment during obstetric emergencies of non-obstetric nursing staff to assist midwives • Wound care by providers rather than students or patients’ caregivers for postoperative infections • Shorten laboratory investigation lead-times through clear assignment of responsibilities • Reviews of patient charts by senior staff and feedback to providers to improve good charting practices • Reassign sterilization to theater nurses exclusively and provide training and monitoring by nurse supervisors
Health systems	*Personnel* • Shortage of skilled staff at some rural health centers • Insufficient provider-to-patient ratio • Poor retention of medical doctors at adjacent district hospitals *Communication/managerial* • Absence of radio or other means of communication between health centers and hospital • Ambiguity of provider work schedules and shift coverage • No backup provider schedule for evacuation emergencies requiring nurse accompaniment • Health centers with no motorcycle ambulance or other means of transportation • Delayed ambulance or delayed referral *Availability of health services* • Lack of basic emergency obstetric and neonatal care (BEmONC) services in peripheral health centers due to lack of trained staff and/or equipment • Absence of sufficiently staffed district hospitals in two adjacent districts *Hospital resources* • Blood product stock-outs • Limited oxygen supply and pulse oximetry • Inadequate number of manual vacuum extractor sets • Inoperable or insufficient anesthetic equipment • Power outages and lack of generator backup for labor and delivery ward • Traditional birth attendant-related delays	• Increase number of obstetric care providers (nurses, midwives, birth attendants) • Training of personnel to identify and respond to obstetric emergencies and scheduling backup staff in such cases • Create incentives for medical doctor retention by improving the work environment • Establish communication channels with 24/7 monitoring at the hospital level • Assign a member of staff to oversee scheduling and disseminate schedules 5 days prior to the start of each month by posting in workspaces • Establish a schedule for nurses assigned to emergency transfers to level-one center • Training and provision of BEmONC equipment for rural health center • Establish a system for recording blood stock-outs and petitioning to provincial Ministry of Health to ensure steady inventories • Lobby MOH and/or donors for additional equipment • Engagement and training of traditional birth attendants
Accessibility	• Long distances from patient village to health center • No district hospital in adjacent districts • Poor road conditions and lack of reliable transport • Ambulance not available to transport patient from health center to hospital • Water transport for island-dwelling patients unreliable	• Infrastructure and transportation improvements by the government • Increase availability of ambulances and emergency boat transport
Patient factors	• Poor antenatal care clinic attendance and late booking • Delay in seeking care • Preference for home delivery • Prioritization of ethnomedical care over biomedical care	• Conduct sensitization activities in the local communities on issue related to health-seeking behavior, home delivery, use of herbal medicines, and antenatal care clinic attendance

### Maternal mortality and obstetric care 2007–2011

**Fig. 2:**
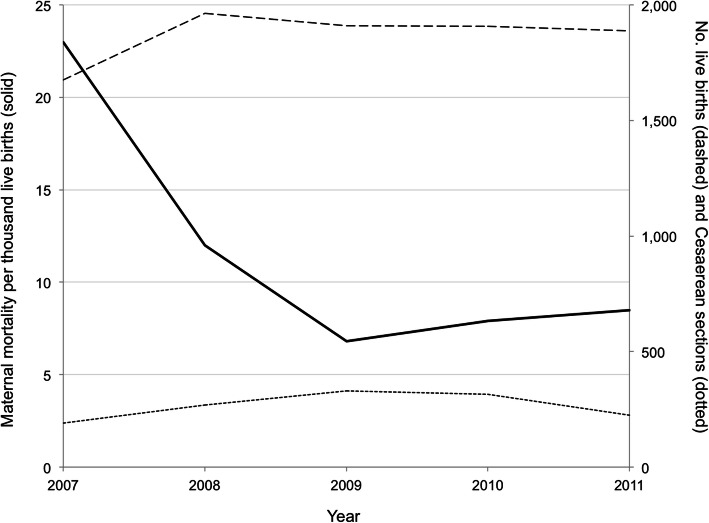
Maternal mortality (solid line) declined with implementation of maternal death reviews in 2007 at a rural district hospital in Zambia, while the number of live births (dashed line) and Cesarean sections (dotted line) remained similar over time

Comparisons across the baseline, early, and late post-intervention period showed a significant decrease in overall in-hospital maternal mortality (Fig. 2). Maternal mortality declined from 23 ± 5.6 per thousand live births in 2007 to 8 ± 1.3 per thousand in 2010–11 (*P* < 0.01). Over the same period, case fatality for puerperal sepsis and uterine rupture decreased from 63 and 32% to 10 and 9%, respectively (P < 0.01) but there was no statistically significant change for postpartum hemorrhage or eclampsia (Table 3). None of the obstetric care metrics showed statistically significant differences between the early and late phases of implementation of MDR committee reviews (Table 4). Trends were observed in fewer transport delays (55% vs. 37%, *P* = 0.18), reduction of missed diagnoses (19% vs. 7%, *P* = 0.11) and medication and supply shortages (8% vs. 0%, *P* = 0.10).

**Table 3:** Maternal mortality indicators

Condition	2007			2010–11			*P* value
	No. deaths	No. cases	CFR (%)	No. deaths	No. cases	CFR (%)	
Postpartum hemorrhage	4	20	20	9	39	23	0.99
Puerperal sepsis	10	16	63	6	63	10	< 0.01
Eclampsia	3	15	20	1	22	5	0.28
Uterine rupture	9	28	32	5	58	9	0.01

*CFR* case fatality ratio. *P* values were calculated by Pearson’s χ^2^ or Fisher’s exact test

**Table 4:** Proportion of maternal deaths with actionable barriers to care, comparing early and late post-intervention periods

Factor	Prevalence, % (no.)			*P* value
	2008–09 *n* = 36		2010–11 *n* = 31	
High risk pregnancy without available expertise	88 (15)		79 (11)	0.40
Delayed transport to facility	55 (18)		37 (10)	0.17
No preliminary care prior to transfer	78 (25)		85 (22)	0.53
Delay in obstetric care after arrival	11 (4)		10 (3)	0.84
Missed or delayed diagnosis	19 (7)		7 (2)	0.11
Partograph use and interpretation error	0 (0)		50 (2)	0.47
Oxytocin administration error	0 (0)		50 (2)	0.16
Magnesium sulfate not given when indicated	20 (1)		0 (0)	0.83
Antibiotic prophylaxis not given when indicated	33 (3)		50 (2)	0.51
Medication and supplies shortage	8 (3)		0 (0)	0.10

*P* values were calculated by Pearson’s χ^2^ or Fisher’s exact test. Denominators were the total number of cases for which a given factor was clinically relevant

### Antenatal clinic attendance and screening practices

One in four mothers who died did not attend antenatal care clinic, and those who did attend did not always receive recommended care. Issues included missing or incomplete screening for high-risk conditions (e.g. pre-eclampsia, anemia), no vital signs recorded, and no planned hospital delivery for complicated pregnancies when hospital delivery was indicated. Initiation of antiretroviral therapy for antenatal clinic patients who screened positive for HIV infection was sometimes delayed or not done due to factors such as unavailable or late testing for CD4 cell count.

### Issues at referring health clinics and referring hospitals

Pre-referral emergency treatment was often not available or not provided at the health centers. Health centers frequently lacked capacity for basic emergency obstetric and neonatal care, and lack of established communication channels between referring centers and the hospital meant missed opportunities for providers to relay pre-transfer instructions and accelerate preparedness at the receiving facility. Patients were at times referred from remote villages in adjacent districts that either had no district hospital, or no physician available at the district hospital.

### Patient preference for home delivery and ethnomedical care

Patient-associated factors included preference for home delivery with traditional birth attendants and reliance on ethnomedical remedies. These and other patient factors contributed to reduced access to biomedical services (phase 1 delay).

### Transportation to the hospital

Limited transportation compounded by long distances from the home to health center, or health center to hospital, caused access and treatment delays (phase 2 or 3 delays). Ambulance transport was delayed in 47% of cases, and 10% of women had to walk due to lack of transportation. A single ambulance was stationed at the district hospital, and its effective deployment was hindered in part due to the absence of official communication channels between health centers and the hospital. Because there was only one ambulance, delays occurred when two or more cases from different health centers required emergency transfer simultaneously. When the ambulance was unavailable, patients and their families, who were largely economically impoverished, were responsible for identifying and funding transportation on their own. Adding to these challenges were poor road conditions and the long distance, up to 300 km, required to travel to reach the hospital from some referral centers. In addition, the hospital serves two island villages; boat transport is expensive and can take several hours to arrange.

### Human resources, essential supplies, and diagnostic evaluation

Once patients arrived at the district hospital, delays in escalation of care (phase 3 delay) occurred when personnel were unavailable and essential resources were not on hand. Understaffing hampered patient care at both the health center and district hospital level, and ambiguity in provider work schedules led to delays and gaps in communication. Stock-outs of blood for transfusion, medications, and essential equipment (e.g. oxygen, vacuum extractor) created additional challenges. Incomplete clinical evaluation including errors in partograph operation and interpretation led to late or missed diagnoses, which in turn delayed escalation of care (phase 3 delay). This translated to clinical interventions such as antibiotic prophylaxis, magnesium sulfate, and oxytocin being delayed, forewent, or used outside of the standard of care.

## Discussion

We conducted a mixed methods convergent study of an MDR program in a rural Zambian hospital to evaluate its impact on inpatient maternal mortality. We hypothesized that health system improvements and other changes affected by MDR implementation would lead to reductions in hospital-based maternal deaths and high-risk features of cases. Our results showed fewer inpatient maternal deaths following MDR implementation, identified contributory factors to maternal mortality, highlighted inherent limitations to the program, and captured general and context-specific recommendations rendered by the MDR committee.

Maternal deaths were 35% lower after MDR was implemented compared to the period before its implementation, similar to results of other before-and-after studies of MDR in sub-Saharan Africa [11, 12]. Inpatient maternal deaths fell from 23 per thousand live births to 8 per thousand. Note that the current study was restricted to inpatient deliveries at a district-level referral hospital, whereas public health statistics cited elsewhere in this report included deliveries at local health centers where the large majority of live births occur, which are typically uncomplicated and result in far fewer deaths.

MDR programs have previously been linked to improvements in maternal outcomes, and prior studies offer insights as to how. In an extensive review, Ivers et al. found slight but significant improvements in professional practice and health outcomes after the introduction of audit- and feedback-based approaches [27]. MDR programs have led to shorter wait times, lower rates of severe complications (e.g. uterine rupture), and higher attainment of the six components of basic emergency obstetric care while organizational restructuring and resource mobilization spurred by MDR-guided feedback has led to lower overall maternal mortality [10, 14–16, 28, 29].

In our series of 106 maternal deaths, the major direct causes were postpartum hemorrhage (21%), puerperal sepsis (19%), uterine rupture (16%), and eclampsia (9%) while the two most common secondary causes were HIV (15%) and malaria (8%). These findings resemble contemporaneous maternal mortality statistics elsewhere in Zambia and sub-Saharan Africa; leading causes of maternal death nationally in Zambiawere postpartum hemorrhage (34%), puerperal sepsis (13%), obstructed labor/uterine rupture (8%), and eclampsia (5%) with 10–11% attributed to each HIV and malaria [21, 22, 30]. The relatively low prevalence of malaria in the sample was somewhat surprising; Nchelenge District is among the most malarious areas in Zambia [31, 32] and although intermittent presumptive treatment of malaria in pregnancy is indicated according to national guidelines, uptake in the community remains low overall [33]. The apparent discrepancy appears to be explained by the fact that malaria in pregnancy is most life-threatening in primigravidae [34], whereas most of the mothers in our study (76%) were multiparous.

The prevailing decline in maternal deaths was driven by lower fatality from puerperal sepsis and uterine rupture. There was also an apparent fall in case fatality from eclampsia (from 20 to 5%) although the number of cases in the post period (*n* = 1) limited statistical power to detect a significant difference. Strikingly, however, case fatality for postpartum hemorrhage did not change. Postpartum hemorrhage was just as likely to be fatal after MDR was implemented as before, with one in five cases ending in the mother’s death.

In Zambia and across sub-Saharan Africa, postpartum hemorrhage is the most common cause of maternal mortality [22, 30], so the apparent ineffectiveness of MDR to lessen its toll warrants special attention. Postpartum hemorrhage requires prompt treatment, therefore delays in reaching a first-level health facility (e.g. due to distance or delayed care-seeking) or second-level facility (e.g. due to delayed or unavailable ambulance) contribute to poor outcomes [35]. The inefficacy of MDR to reduce postpartum hemorrhage deaths signals a need for material investments and capacity building beyond MDR. When a mother who recently gave birth or underwent a Cesarean section has major internal bleeding, prompt diagnosis and rapid response by a skilled surgical team are necessary but insufficient if, for example, the mother cannot reach a facility in time, or if the facility has no blood for transfusion. Policies with monetary backing that build capacity for basic emergency obstetric services, ensure that blood banks are stocked, and adequately equip operating theaters are equally crucial and should follow hand-in-hand with the upskilling of healthcare workers that MDR accommodates [36].

The causes of delay, challenges to care, and medical errors identified by the MDR committee encompassed individual, institutional, and structural elements that echoed findings from the earliest MDR program assessments done as far away and far back as England and Wales in the 1950s [8] to more recent accounts in neighboring sub-Saharan Africa countries [12–17]. Prior studies of MDR programs in sub-Saharan Africa documented many of the same factors contributing to maternal death, and similarly point to root causes that are structural, beyond the sweep of MDR committees or their regional governments [20, 37, 38]. These factors can be considered according to the Thaddeus paradigm of delays in care (e.g. transportation delays, delayed care-seeking, late diagnosis leading to an in-hospital delay) [23] and they are one and the same as the structural barriers that accompany unalleviated poverty: shortages of life-saving supplies and skilled staff, competing priorities that vie for scarce resources, whole districts without hospitals.

Many of the mothers in our study (39%) were referred from outside the district, and most (76%) received at least some antenatal care, highlighting the opportunity for upstream maternal health interventions. Reducing maternal deaths due to complications of obstructed labor and eclampsia in particular demands skilled antenatal care and early diagnosis and treatment at pre-referral centers [14, 39]. Our study also described patient preferences for ethnomedical care and home delivery, which carry previously well-characterized risks due to inadequate pharmacotherapies and unskilled management of labor complications [28, 40, 41]. Logistical barriers due to transportation were prevalent in the study, which have an extensive literature documenting their contribution to maternal deaths by delaying urgently needed care [42, 43]. These upstream factors anticipate an opportunity for maternal health and patient education-based interventions beyond hospital-based MDR [44].

Recommendations proposed by the MDR committee ranged from pragmatic solutions that could be enacted at the point of care, to farther-reaching appeals to the district, provincial, and national health offices. More proximal recommendations sought to address staff performance, coordination of care, and delegation of responsibility, in line with interventions previously shown to reduce maternal deaths [10, 16]. These included recommendations to train staff on current guidelines, improve documentation, promote teamwork and clear communication, devise clear work schedules that are disseminated effectively, and to assign skilled tasks to the appropriate provider (e.g. wound dressing changes, sterilization of surgical equipment). Community-level recommendations included engaging traditional birth attendants, for which there is mixed evidence to support reductions in maternal mortality [45–47], and community sensitization via radio announcements to increase antenatal care clinic attendance.

Several of the MDR committee recommendations implicated chronic understaffing. Understaffing is associated with lower quality of care and higher maternal mortality, and was previously identified as a major barrier to achieving the Millennium Development Goals [48, 49]. MDR recommendations instructed personnel to take up duties outside their typical purviews, which can lead to worse maternal health outcomes and job dissatisfaction on the part of providers [48]. Midwives were recommended for post-operative wound care, nurses from non-obstetric wards for obstetric emergencies, surgical nurses for supervising sterilization procedures typically performed by trained environmental staff, and laboratory technicians for ensuring blood supplies despite having no capacity for blood collection, atop frequent stock-outs in the nearest blood bank located 240 km away. Finally, all of the MDR committee recommendations have a prerequisite for effective leadership at all levels, a recognized necessity for the success of any maternal health intervention [50]. From staff performance (e.g. adherence to guidelines, appropriate partograph use) to the delegation of duties and fostering channels of communication, several of the contributing factors and the recommendations they spurred can be traced to, or rest upon, good leadership.

There are limitations to this study that are inherent to its before-and-after design, such as the lack of rigorous control group or ability to show causation, and others that are particular to the study sample and context. Anecdotally, there were no obvious socioeconomic or other secular trends over the comparison periods to account for improved outcomes, although we did not measure indices of poverty, literacy, education, or other potential explanatory factors. Similarly, we cannot discount the possibility of decreased referrals of complicated cases resulting in a shift of maternal deaths from the hospital to peripheral centers, although casual observations from the field did not support this. Despite fewer maternal deaths seen in the before-and-after analysis, we found no clear patterns in the before-and-after analysis of potential explanatory factors, outlined in Table 4, to suggest the drivers of the change. There were downward trends in transportation delays, missed diagnoses, and medication and supply stock-outs. The analysis was hindered by the lack of a more stringent comparator groups (e.g. mothers with the same diagnoses who survived) and small sample size. Our study included only mothers who presented or were transferred to the district hospital, therefore pre-hospital deaths were not captured, and unlike some other MDR programs ours did not include near misses.

## Conclusion

Our analysis of more than one hundred MDR reports portrays the complex web of health care delivery that interlinks mothers, health providers, clinics and hospitals, local communities, policymakers, and budgetary officers. In Nchelenge, their interrelationships and commitment to the common goal of reducing maternal mortality are confounded by economic underdevelopment and its attendant hardships. Infrastructure that is hard-worn or nonexistent, such as empty blood banks, impassable roads or want of vehicle, and shortages of essential equipment impedes the efforts of MDR committees.

This study contributes evidence of MDR’s effectiveness in reducing maternal deaths, but also unveiled a critical gap. While maternal deaths from three of the top four causes fell after implementation of MDR, deaths due to postpartum hemorrhage—the most common cause of maternal death in Zambia and the rest of sub-Saharan Africa—remained unchanged. Future research should focus on causal analysis and interventional studies to better understand and ultimately close this gap. MDR programs are impactful but alone are insufficient to eliminate the vast disparities in obstetric care between low and high income countries. In Zambia and similar resource-deprived settings, progress must continue to rely on financial and material provisioning by global aid, regional and national governments, and other holders of the purse strings while fuller economic development awaits realization.

## Data Availability

The datasets used/or analysed during the current study can be made available from the corresponding author upon reasonable request.

## **Appendix 1**

**Table 5:** Data items extracted from maternal death review reports

Category	Factors
General information	Date and cause of death
	Age, parity, and gravidity
	District of provenance
Clinical history	History of previous Caesarean section
	Antenatal care attendance
Features of clinical case	Place of delivery (home, health facility)
	Use of traditional medicine
	Means of transport to health facility
	Missed diagnosis
	Partograph use and interpretation
	Oxytocin use
	Magnesium sulfate administration
	Antibiotic prophylaxis use
Delays in care	Phase 1 (delay in care seeking)
	Phase 2 (delay due to geographical location, etc.)
	Phase 3 (delay in provision of care)
Contributive factors and recommendations	Standards of care Health systems organization and management Accessibility to care Patient factors

## Abbreviations

CFR: Case fatality ratio
MDR: Maternal death review
